# Applying technology to promote sexual and reproductive health and prevent gender based violence for adolescents in low and middle-income countries: digital health strategies synthesis from an umbrella review

**DOI:** 10.1186/s12913-022-08673-0

**Published:** 2022-11-19

**Authors:** Keng-Yen Huang, Manasi Kumar, Sabrina Cheng, Anya Elena Urcuyo, Paul Macharia

**Affiliations:** 1 Department of Population Health, New York University School of Medicine, 227 East 30Th Street, 7Th Floor, New York, NY 10016 USA; 2 Brain and Mind Institute, Aga Khan University, Nairobi, Kenya; 3 Department of Psychology, Florida International University, Miami, USA; 4 Consulting in Health Informatics, Nairobi, Kenya

**Keywords:** Gender-based violence, Intimate partner violence, Sexual and reproductive health, Low-and-middle-income countries, mHealth, Digital health, Adolescents Development, Review

## Abstract

**Aim:**

Adolescents in low-and-middle-income countries (LMICs) are facing numerous developmental, sexual and reproductive health (SRH) challenges including exposure to multidimensional violence. Gender-based violence (GBV) specifically intimate partner violence (IPV) are both highly prevalent in LMICs and are strongly linked with poor SRH outcomes. However, GBV and IPV interventions have not yet been adequately integrated in SRH due to individual, social, cultural, service, and resource barriers. To promote long-term SRH, a more holistic approach that integrates GBV and IPV, and adolescent development needs is imperative. Digital health has the potential to address multiple service setup, provision, and addressing access barriers through designing and providing integrated SRH care. However, there are no guidelines for an integrated digital SRH and development promotion for adolescents in LMICs.

**Methods:**

An *umbrella review* was conducted to synthesize evidence in three inter-related areas of digital health intervention literature: (i) SRH, (ii) GBV specifically IPV as a subset, and (iii) adolescent development and health promotion. We first synthesize findings for each area of research, then further analyze the implications and opportunities to inform approaches to develop an integrated intervention that can holistically address multiple SRH needs of adolescents in LMICs. Articles published in English, between 2010 and 2020, and from PubMed were included.

**Results:**

Seventeen review articles met our review inclusion criterion. Our primary finding is that application of digital health strategies for adolescent SRH promotion is highly feasible and acceptable. Although effectiveness evidence is insufficient to make strong recommendations for interventions and best practices suggestions, some user-centered design guidelines have been proposed for web-based health information and health application design for adolescent use. Additionally, several digital health strategies have also been identified that can be used to further develop integrated GBV-IPV-SRH-informed services to improve adolescent health outcomes. We generated several recommendations and strategies to guide future digital based SRH promotion research from our review.

**Conclusions:**

Rigorous research that focuses on intervention effectiveness testing using a combination of digital health strategies and standardized albeit contextualized outcome measures would be important. Methodological improvement such as adoption of longitudinal experimental design will be crucial in generating evidence-based intervention and practice guidelines for adolescents in LMICs.

## Background

### Sexual and reproductive health needs and service gaps for adolescents in LMICs

Sexual and reproductive health (SRH) is defined as “a state of physical, emotional, mental, and social wellbeing in all matters relating to sexuality, the reproductive system, and its processes” [1]. SRH services generally cover 9 topic areas, including *(i) contraception/pregnancy prevention, (ii) HIV/AIDS, (iii) sexually transmitted infections (STIs), (iv) abortion, (v) antenatal care/delivery/postnatal care, (vi) reproductive tract infections, (vii) infertility, (viii) sexuality, and (ix) partner violence (including violence against women) and related practices (e.g., female genital mutilation)* [2]. As part of normal development, adolescents face important decisions related to their SRH, sexual relationships, sexual identity, and sexual activities. They also experience rapid physical (puberty, body shape), cognitive (moral concepts, complex abstract thinking; self-identity), and psychosocial development (peer identification, heterosexual peer interest, sexual orientation, formation of intimate relationship). The domains of SRH and overall adolescent development are interrelated. Because puberty, body image, sexual identity, and intimate partnershipsare part of normative adolescent development, constructing healthy relationships, gaining timely knowledge and practice during the adolescent period can shape future healthy SRH [3, 4]. Because of differences in developmental characteristics between adolescents and adults, health promotion strategies should also be differentiated for adolescents [5]. To effectively promote SRH in adolescents, comprehensive care that includes essential adolescent preventive services (i.e., education, adequate material supply/support, welfare planning) and simultaneously use targeted strategies focusing on adolescents’ unique developmental characteristics (in biological, social, and cognitive domains) and living contexts would matter [6, 7].

Gender-based violence (GBV), an important aspect of SRH, is a highly prevalent problem of public health and social significance in adolescents and young adults in LMICs [8–10], and it has a debilitating impact on SRH outcomes. However, adolescent GBV prevention tends to be regimented to adult models that lack developmental contextualization. GBV includes intimate partner violence (IPV), non-partner perpetrated sexual violence, child abuse, female genital mutilation/cutting (FGMC), and child marriage. In this paper we draw attention to the broad practices associated with GBV but keep IPV as an example of most rampant form of violence in youth. An estimated 32–38% of women aged 15–49 years in Sub-Saharan Africa (SSA) experience IPV (one of the most common forms of GBV) in the past year (38–50% lifetime prevalence) [8]. In the Asia Pacific, 8–32% of women aged 18–49 years and 10–34% of men reported IPV experience in the past year, with a higher lifetime prevalence of IPV for men [33–80%] than for women [28–67%] [9]. A trans-regional study based on 46 countries also reported an overall higher prevalence of IPV in younger women (15–19 years old) than the older women (20–34 or 35–49) [10]. High prevalence of GBV specifically IPV is associated with a host of negative SRH outcomes, including high risks for HIV/STIs, abortion, risky sexual behaviors, number of sexual partners, low use of contraception/STI prevention, unintended pregnancies, sexual disempowerment (e.g., sexual communication, relationship equity), and poor mental health [11]. Therefore, both GBV and a strong focus within it on IPV prevention should be deemed as an important area of SRH care in LMIC contexts [11].

Challenges in addressing violence within SRH service context in LMICs can be attributed to complex, multilayered risk factors, limited intervention and implementation evidence, and health policy -and system-level barriers. Specifically, GBV and IPV can be related to a spectrum of *individual risks* (such as early childhood adversities, multiple traumatic experiences related to GBV, personal characteristics), and *sexual partner risks* (such as partners’ gender stereotypical views, substance abuse history, unemployment, and infidelity) [9]. Additionally, these may be related to *inequitable socio-cultural norms* (such as norm experience in witnessed GBV and IPV well before puberty and first dating relationship; and difficulty in disclosing IPV and GBV given culture stigma, shame, and associated fear and guilt). Service gaps can also be due to *limited intervention and implementation evidence* and a *lack of health policy guidelines*. Existing adolescent SRH interventions in LMICs have mostly focused on topics related to contraception, family planning, abortion, teen pregnancy, sexually transmitted infections (STIs), and maternal depression [12–15], and do not adequately address the root causes [11]. Policy guidelines related to safe, and appropriate IPV, GBV, and SRH knowledge dissemination are known to be deficient [16, 17]. Solutions toward GBV management are generally not adolescent-sensitive or consider socio-cultural nuances that might impact how GBV is practiced and how it shapes adolescent developmental experience [11, 17, 18], In part due to lack of knowledge about IPV, training, awareness and prevention skills around service implementation in providers [6, 19] this tends to remain under addressed. Most existing GBV interventions in LMICs focus on women and adults [20, 21], are provided within social welfare or legal services, and are not integrated within SRH services [22–24]. Therefore, the burden associated with GBV specifically around IPV continues to adversely impact LMIC populations’ SRH outcomes [7, 25].

### Moving towards developing an adolescent-centered and integrated SRH service with use of technology

The Lancet Commission on Adolescent Health and Wellbeing has called for new ways of adolescent health promotion that consider multiple adolescent health and developmental needs and living experience [4]. To effectively promote adolescents’ SRH in LMICs, it is critical to consider a comprehensive integrated approach of service development *that addresses developmental, GBV targeting IPV, and service needs that address sexual health promotion* [6, 19]. To help the field move towards this integrated approach in the LMIC context, our contribution here is to synthesize evidence in three interrelated areas (SRH, GBV/IPV, adolescent development), and generate lessons to inform future high-impact intervention program development. Given that technology has the potential to offer new cost-effective and longer-term sustainable solutions to address these gaps, our review, and integrated SRH service strategies focus on technology approaches of interventions. In technology and health research, the terms *digital health* and *eHealth* are often used interchangeably*. eHealth*, a broader term used in 2010s defined as the use of mobile health (mHealth), information and communication technology (ICT), computer science, and data to support informed decision-making by individuals, the health workforce, and the health system, to strengthen resilience to disease and improve health and wellness for all [26]. *Digital health* is a term used much broadly after 2018–2019 when WHO introducing new guidelines. The new term digital health considers art and science aspects of technology (e.g., visual, personalize, communication forms of presentation) [27, 28]. The applications of technology in health holds promise. Digital health can be used for screening, health education, addressing social-cultural and privacy challenges, offering tailored services to individuals, and providing self-help support. It can improve service efficiency, improve coordination, linkages, and be offered easily by non-professionals. Based on these recommendations, it strongly presents itself as a low-cost sustainable model [29, 30] to bridge this gap. A review study based on 66 articles covering Sub-Saharan Africa (SSA) found that eHealth has been applied to address a range of health conditions. These include infectious diseases (malaria, HIV/AIDS), oral health, infant health, maternal health (antenatal/postnatal care, postpartum hemorrhage), non-communicable diseases (cervical cancer, blood pressure), and some areas of mental health (such as depression care) [31]. Most digital health strategies were designed for disease control and prevention, population health monitoring, information provision for treatment/prevention, data acquisition, and patient records management, diagnosis, training/recruiting/retaining health professionals, or decision-making/referrals [31].

In applications of digital health for young people, WHO has proposed a youth-centered digital health intervention framework for planning, development, and implementing solutions for young people [5]. A body of research has also documented the effectiveness of using digital health to facilitate *youth self-health management approaches* (e.g., chronic illness, mental health) [32–35] and *improved behavioral support* to promote youths’ mental health [36–41]. Although there is a high percentage of digital device ownership, an emerging trend in LMICs to seek health information from the internet (42–51%) [42–44], and the potential of using digital Health to address multiple adolescent health barriers [45], digital health has not been systematically studied in adolescent IPV and GBV intervention for adolescents in LMICs [46, 47].

New implementation science methodologies, like intervention integration, can be applied to systematically adapt and combine existing evidence-based interventions (EBIs) to align them with population needs (interrelated SRH issues), new populations (e.g., adolescents), or implementation contexts (e.g., targeting high GBV contexts), and help the field to develop, select, or tailor implementation strategies to increase adoption, implementation, and sustainability [47, 48]. The combination use of digital health and implementation strategies/methodologies offers new opportunities to create novel approaches of integrated interventions to disrupt the traditional model of SRH access and care to allow for user-friendly solutions. Therefore, in our digital health review, we focus on synthesizing relevant digital health strategies as well as implementation strategies.

### Umbrella review aims

As a first step to inform the development of integrated digital health SRH intervention and services, it is critical to first understand current technology applications and digital health strategies applied in inter-related areas of adolescent health to better identify opportunities, leverage existing technology solutions to put in place adolescent engaged integrated SRH care. Numerous digital health review studies have been conducted, but these in our assessment lack a holistic approach for a well-rounded adolescent SRH promotion. This paper is an umbrella review [49] purporting to summarize three inter-related areas of digital health literature to inform the development of adolescent centered integrated SRH services. This review is not intended to replicate the existing review studies. Instead, we aim to provide an overview and summary for each area of literature first; but most importantly, to identify opportunities and strategies for integrations.

Thus, we focus our review to find answers for two broader areas of questions:

- *What are the digital health intervention designs, effective digital health implementation strategies, and impact evidence for (i) promoting adolescent SRH, (ii) preventing IPV and GBV, and (iii) promoting adolescent development?* As described above, multilevel risks/factors might contribute to poor health in adolescents (described above). We focus on synthesizing literature in digital intervention contents that have been applied in addressing SRH and GBV/IPV needs.
- *What are the potential implementation strategies that can be leveraged from the existing three areas of digital health EBI research for furthering adolescent centered, integrated SRH services*?

## Methods

### Literature review approach

An umbrella review is developed, which provides a concise and focused synthesis addressing our research questions mentioned above [49]. As described above, a body of digital health research has been published in SRH, GBV, and adolescent development, t*his paper focuses on reviewing existing review articles* [49] *and synthesized high-level key findings* from the literature. We included literature from both high-income countries and LMICs because of limited adolescent-focused digital health research from LMICs, and the potential to transport lessons learned to LMICs. *To answer the first research question* (identifying effective digital health intervention designs and implementation strategies), the PICOS evidence-report framework (suggest reporting Population targeted, digital health Intervention characteristics/design strategies, Comparison, Outcome, and Study type of the targeted studies) was applied to summarize the literature [50, 51]. Considering digital health research is an emerging field and we might not find many systematic review articles, we considered both systematic and less formal review articles. Systematic review, meta-analysis, scoping review, qualitative review, and mixed-methods types of review articles are included. In addition, review articles published in English, between 2010 and 2020, from PubMed (one of the largest databases that cover most health intervention and implementation research), and those that targeted adolescents were included. We limited the articles after 2010 because digital health research prior to 2010 was mainly mobile/cell-phone based and relied on basic text/messaging system [52], which is less relevant to the applications that used today. Our singular focus here is to conduct a comprehensive systematic review for three included areas of research. Table 1 describes the article search terms and strategies applied in identifying articles in each research area. *To answer the second question* (strategies for integrating GBV-SRH-developmental service for adolescents), we based on a team consensus approach to further identify research and practice strategies from our three areas of review findings that can be integrated to better develop integrated SRH care. We summarize a set of practice recommendations for moving towards adolescent-centered, integrated SRH services.

**Table 1:** Article search terms and strategies in three areas of eHealth

Area of digital health	Review Articles Search Strategies	Review Article Selection
1. SRH	(Sexual Health or Reproductive Health) and (mHealth or eHealth) and (Adolescent), filters: in the last 10 years	142 articles 7 review articles identified for this review [23, 53–58]
2. GBV	(IPV or GBV or Dating Violence) and (mHealth or eHealth), filters: in the last 10 years	38 articles 4 review articles identified for this review [59–62]
3. Adolescent Development, Behavioral Health	(Adolescent Development) and (mHealth or eHealth) and (Adolescent), filters: in the last 10 years	483 articles 6 review articles identified for this review [63–68]

***Note.*** The search terms ‘eHealth’ and ‘mHealth’ were based on WHO’s recommendations for technology-based health research between 2010 and 2019. For adolescent development, we focused on eHealth/mHealth review articles that examined broader applications of technology in promoting adolescents’ development, social, emotional, and general physical health literacy/skills/competency, as well eHealth/mHealth review articles focused on design strategies to promote engagement in using technology solutions in general adolescent health. We excluded articles focused on mental disorders, chronic diseases, or medical interventions gave the special needs (anxiety and depression, suicidal intervention, diet/physical activity/weight management, tuberculosis treatment). For GBV literature, we included ‘IPV’ and ‘dating violence’ search terms because these terms are more commonly used in the adolescent literature. In addition, we included review studies targeting any age group because no digital health review paper targets adolescents specifically (most studies include small subgroups of adolescents, and do not examine age differential impacts)

## Results

A total of 17 review articles fit our inclusion criteria for this review (see Table 1). These included 7 review articles from the SRH digital health category, 4 review articles from the GBV including IPV digital health category, and 6 review articles from the adolescent development and behavioral health category. Tables 2, 3, and 4 summarize the evidence for each area of literature using the PICOS framework [50, 51]. *Based on the results from each table, we further synthesized and organized digital health findings in six areas: 1) targeted population/users characteristic; 2) topics covered and characteristic of the digital health intervention; 3) digital health strategies and implementation strategies applied; 4) the feasibility of the tested digital health interventions; 5) efficacy/effectiveness evidence and recommendations; 6) gaps in the digital health interventions. We summarize these six areas of findings for each review area*.

**Table 2:** eHealth for adolescent SRH [23, 53–58]

Article	Aim & Methods	Population (P)	Intervention (I)	Comparison (C)	Outcome (O)
Chen et al. (2016) [54]	**Aim:** Review mobile apps for pregnancy prevention used in adolescents and young adults (AYAs) to generate guidelines on best practices **Methods** • 22 free English smartphone Apps in the Apple App Store (n = 13) and Google Play (n = 9) (identified in 2015) were included	• P: AYAs • Countries: most targeted the US/Canada (k = 9) and UK (k = 7). Some apps focused on LMICs (k = 3): Kenya, Pakistan • Almost all (k = 21) apps provided info relevant for both males and females • Almost half (k = 10) of the apps were downloaded less than 1000 times, 4 were downloaded 1000 to 10,000 times, and 8 apps did not have info	• ***App characteristics:*** in app store categories: Health & Fitness (k = 8), Education (k = 5), Lifestyle (k = 6), Reference (k = 1), Medical (k = 1), and Entertainment (k = 1). Most cited credible info from a reputable public health source like CDC (k = 17) and provided SRH education (k = 19). Many also provided linkage to care (k = 13) and counseling or support (k = 12) • ***User Interface features***: the most common features were for clinic and service locators (k = 12). < 50% (k = 9) had GPS capabilities. Less than a quarter offered entertainment, gamification, or communication features (k = 5, 5, and 4, respectively) • ***E-SRH TX topics:*** most include unintended pregnancy prevention (20/22 condom use info, 14/22 contraception use info, 14/22 service location); STI (19/22), provide info about or refer for abortion services (12/22); < 50% apps describe or counsel on abusive relationships/IPV (10/22), alcohol/ substance abuse (5/22), refer for pregnancy testing (10/22), mentioned confidentiality/privacy (10/22), state app is not a replacement for professional medical advice (4/22)	• ***High quality Apps***: 3 are recommended- (i) *my choice by PPT* (includes 5 of the 8 best practices and one promising practice. (ii) *Safe Sex Tips* (includes 5 best practices but no promising practices), (iii) *Get S.M.A.R.T.* (includes 4 best practices and 2 promising practices) • **Apps with high # SRH features**: 6 Apps included 4 or more SRH features. *my choice by PPT* includes the highest SRH features (6/7)	• Evidence not examined • **Generate 8 best practice guidelines** for developing mobile apps for AYA pregnancy prevention (*Mobile Criteria for Adolescent Pregnancy Prevention; mCAPP)* • ***How the best pregnancy prevention practices guideline integrated in the Apps***: among the 8 guideline, the most commonly implemented best practice was the *provision of information* on how to use contraceptives to prevent pregnancy (15/22), followed by provision of *accurate information on pregnancy risk of sexual behaviors* (13/22); information on *SRH communication, negotiation, or refusal skills* (10/22); and the *use of persuasive language* around contraceptive use (9/22)
Gabarron et al. (2016) [55]	**Aim:** Scoping review of literature on the use of online social media for sexual health promotion & STIs prevention **Methods** • 51 articles published between 2020–2015 were included (4 RCT, 39 non-randomized, 8 observational studies)	• P: AYAs. 59% of studies (k = 30) focused on youth or young people (aged 11–29 years), 11 (22%) focus on adults • Countries: Most were in developed countries (US, Canada, Europe, Australia, New Zealand), 10 in LMICs (Nigeria, Ghana, Pakistan, Peru); and in 79 countries	• **Technology/Social media platforms used**: Facebook, Twitter, YouTube, Instagram, and Snapchat were used. 86% publications (n = 44) used Facebook • **E-SRH TX topics**: 57% of studies (k = 29) focused on the **‘***general’ sexual health promotion or to increase STI testing*; 29% focused on the *incurable STI, HIV* (k = 15), and 14% addressed *curable STIs* such as chlamydia, syphilis, gonorrhea, or HPV (k = 7) • ***Digital-Strategies***: 23 publications used social media as only strategy to promote sexual health, and 28 publications use social media as resource tools (website, games, on-air component) to support a sexual health promotion	• In 4 RCTs (all *use Facebook*), 2 for STI and 2 for HIV prevention • In 39 non-RCT studies (use *Twitter, YouTube, Facebook, Whatsapp, MySpace*), 5 applied theory (Kelly’s opinion leader model, game-based learning with participatory, Penders’ health promotion, peer education model)	**RCT evidence**: (1) positive association between participation in the Facebook group (↑network ties) and the likelihood of HIV testing, follow-up for test results, and participation in group discussion; (2) 2-month short-term, but not 6-month longer term TX effect on condom use and sex acts protection. (3) no impact on HPV vaccination rate **Non-RCT evidence**: Two projects referring to the Kelly’s popular opinion leader model and Pender’s health promotion model, reported positive results regarding an increase in intention to test (43.9%/22.3%) and in intention to use condoms (34.2%/26.2%); 23% reported an increase in condom utilization, and 54% reported a reduction in positive chlamydia cases among 15–17 years olds **Observational study evidence:** social media were reported to be pervasive, and the study participants reacted positively to using new technologies for sexual health promotion or education. In studies with adults, the importance of considering privacy, stigma, and social norms was emphasized, and in this sense, links to social media profiles were not considered to be appealing
L’Engle, et al. (2016) [57]	**Aim**: Systematic review of mobile phone TXs for adolescent SRH **Methods** • 35 articles, representing 28 programs published between 2000 & 2014 were included • 9 RCTs test the impact on SRH (knowledge, sexual behavior, medication adherence, and contraception use, quality of health service delivery, uptake of health screening and treatment services)	• P: Target 10–24 years AYAs both genders • Countries: 7 Countries. 21 US (including Laatino, African American), 4 Australia/New Zealand, 1 Netherlands. 1 Tanzania, 1 Democratic Republic of the Congo)	• **E-SRH TX topics**: 8 focused on *pregnancy*, 4 on *contraceptive method*, 2 on youth assets and *broader pregnancy prevention messaging*, 2 to *pregnant or parenting adolescent*s. *8 on STI*s (STI vaccination, screening, or treatment), 8 on *HIV/AIDS* (prevention or support for HIV-positive youth) • **Mobile phone function used**: Most programs (82%) used text messages (a mobile platform for youth to text SRH questions). Some add mobile phone voice call, and a few use mobile phone videos, email, instant messaging, or multimedia applications • **Digital Strategy used in SRH TXs**: (1) Health promotion campaigns (43%) were the most common purpose of the e-SRH TXs. These TXs provided a mobile platform for youth to text SRH questions to professionals, allowed adolescents to retrieve on-demand SRH content and offered “push” messaging where SRH content was texted to adolescents on a regular schedule. (2) The mHealth TXs for screening and follow-up service utilization (k = 7) included human papillomavirus (HPV) vaccination text message reminders for follow-up, notification for positive chlamydia and other STI results, and chlamydia screening promotion. Patient adherence to medications or health recommendations was addressed in 7 additional programs that provided text message reminders for taking daily oral contraceptive pills or ART for HIV patients	–	• **High acceptability** for using technology **Impact evidence by focus of TXs** • **Health promotion campaigns with curriculum (**e.g., multimedia + SMS messaging) was association with more optimal sexual health knowledge and behaviors (↑protected sex & STI testing) • S**TI screening/follow-up TXs**: (1) combining text messages with a small financial incentive to encourage screening; (2) SMS to parents or teens yielded higher rates of receiving second and third doses of the HPV vaccine and more timely completion of the HPV vaccine series • **mHealth Provider Counseling**: follow-up mobile phone calls (for reminder or support) improve adherence to HIV TX, but inconsistent or no evidence to support impact on contraceptive behavior, STI rates, or other SRH outcomes • **Text messages on oral contraceptive pill (OCP) use**: individuals received education text messages or video messages were more likely to improve OCP knowledge and continue OCP use overtime; interactive text message provide a helpful tool to identify and respond to adherence challenges
Badawy et al. (2017) [53]	**Aim:** Systematic review of texting & mobile phone app TXs for improving adherence to preventive behaviors **Methods** • 19 experimental or pre-experimental design (11/19 RCTs, 5 user ITT), literature published between 1995–2015 were included • 2–12 months follow up period	• P: Adolescents 12–24 years old • Countries: Most studies were performed in the United States (47%, 9/19), included diverse younger adolescents ≥ 12 and < 18 years (63%, 12/19) • Settings: Most were conducted in a clinical setting (9), others were in university (3), vocational schools (2), summer camp (1), participant’s home (1)	• **SRH & Behavioral TX topics:** The 11 RCTs examined whether texting/mobile phone improve adherence to *sexual health behavio*rs (k = 3), *smoking & alcohol use* (k = 2), and other behaviors (k = 6; weight management, oral hygiene) • **Digital Strategy used:** TXs based on texting or Apps ***(i)Texting TX (15 studies)*:** 10 included texting only, 5 included additional components (2 added in-person training sessions, 2 added WB program, 1 added internet-based curriculum). Text reminders were sent once daily in 7 studies, once or twice weekly in 5 studies. Text reminder directionality was 1-way in 7 studies, 2-way in 8 studies, with emotion icon response in 1 study, and sophisticated tailored algorithm in 3 studies. Appointment reminders were sent at a differing frequency (1 reminder/day in 7 studies; daily for 3 days before in 1 study; customized to patient preference in 5 studies). Text messages were also used as a tool for Education (k = 7), positive reinforcement or personalized feedback (k = 5), goal setting (k = 3), addressing barriers (k = 1), or for incentive/as a reward system for promoting patient engagement (k = 9) *(****ii)Mobile App TXs (5 studies)*:** It has not been used for SRH. 2/5 use Apps to improve fitness adherence behaviors (WhatsApp, Zombies, Run! 5 K Training app)	• Only 32% of studies (k = 6) incorporated a behavioral theoretical framework in design (e.g., Transtheoretical Model, Geser’s Sociological Framework, Health Belief Model and Information Motivation Behavior Model, Health Action Process Approach, Stages Motivational Readiness for Change Model, Addiction Treatment Model)	• In 3 studies related to SRH, texting *did not* improve condom use, drug or alcohol use before last sex (ns), or improve SRH knowledge (e.g., HIV), attitude toward condoms, or reduction in risk behaviors (during intercourse, illegal drugs use). Testing does improve HPV vaccine does 2 and 3 completion • Most studies reported good feasibility with high acceptability and satisfaction. About half of the included studies (42%, 8/19) demonstrated significant improvement in preventive behavior with moderate standardized mean differences • Most studies were low to moderate in quality
Ippoliti, et al. (2017) [56]	**Aim:** Review phone programs for adolescent SRH in LMICs **Methods** • 17 SRH projects were review (include GBV, contraception methods/ knowledge, sexual health, STI info, HIV, family planning; improve provision of medical abortion and post abortion family planning use)	• P: Youth ages 10–24 (in or out of school adolescents) • Countries: 67% Africa (e.g., Nigeria, Senegal, Morocco, Egypt, Cambodia), 26% Eurasia (European & Asia), and 13% Latin American countries	• **SRH TX topics:** The majority of mHealth projects (82%) were for SRH **health promotion** (to facilitate knowledge sharing and behavior change). Other projects (18%) used mHealth as a way to **link users to essential SRH services** (e.g., family planning counseling services, medical abortion and post-abortion care, and HIV care and treatment) • **Phone use Strategies**: Little variation in delivery methods for TX content, as 70% of the projects relied on text messaging to facilitate knowledge sharing and behavior change (e.g., transmit health message on post-abortion care, psychosocial support for cases live in HIV, theory-based role model story narratives, counseling). The remaining TXs (5 projects) employed a mix of informational hotlines, social media/Facebook, and email applications to reach & support their users • **SMS functionalities for knowledge sharing**: (i) *SMS provide a platform for youth to text SRH question* to a health professional*, allow the adolescent to retrieve “on-demand” SRH content* through a question and answer platform, (ii) “*push” SRH messaging (through an information message system)* to adolescent on a regular schedule; *share role model stories* that show common barriers to contraceptive use faced by youth; (iii) *use social media website online forum and mobile apps to disseminate HIV p*revention, treatment, and care info • **Mobile functionalities for providing psychosocial support**: using SMS or Voice-based info hotline to support HIV positive adolescents • **Implement by trained counselors:** train counselors to provide rapid, accurate, and non-judgmental answers, or use a database of answers to frequently asked questions or customized replies	**TX Examples**: • *m-ASSIST* and *Project Khuluma* use SMS to transmit a message on post-abortion care and psychosocial support for HIV youth, respectively*;* • *m4RH* and *mCENAS* use SMS to transmit role model stories about family planning, contraceptive use and decision-making in reproductive age • *Learning about Living* uses Q&A service operates (on SMS & internet- platforms) to provide knowledge and support for sexual health • *ChatSalud* in Nicaragua uses a SMS interactive platform for health promotion (allows users to customize which info to read)	**High acceptability:** SMSs delivered through the mobile platform were reported high acceptability **Limited effectiveness evidence**: RCT studies found (i) m4RH improved family planning knowledge, but not family planning use;(ii) m-ASSIST reduce anxiety and stress for those who receive SMS message and standard care;
Widman, et al. (2019) [58]	**Aim:** Meta-analysis of the literature on technology-based sexual health TX **Methods** • 16 experimental studies (RCT or quasi-experiment), published in English & before May 2017, & included condom use and abstinence as outcomes	• P: Youth ages 13–24. Most study focused on boys and girls (k = 9), but a few studies included only boys (k = 3) or girls (k = 4) • Countries: Most from the US (k = 11)	• **Technology used in the e-SRH TXs**: e-Sexual Health interventions were reviewed (5 programs delivered via computer programs, 2 were through internet websites, 1 through texting, 1 through social media), and nearly half were with more than one method (e.g., internet + email follow up, or texting + email delivery) • **Digital strategies**: TXs tended to use interactive (accept input from the user, k = 12) and tailored (k = 10) e-strategies. The dose of TX varied, range from 1–2 sessions (k = 5) to 7 or more sessions (k = 8)	**Subgroup Comparisons**: Effects did not differ by age, gender, country, intervention dose, interactivity, or program tailoring. However, effects were stronger when assessed with short-term (1–5 months) compared to longer-term (greater than 6 months) follow-ups	**Condom use & abstinence**: There was a small but significant protective effect of technology-based interventions on *condom use* (d = .23, 95% CI [0.12, 0.34]) and *abstinence* (d = .21, 95% CI [0.02, 0.40], p = .027) **Other SRH outcomes**: Compared to control programs, technology-based interventions were also more effective in increasing *sexual health knowledge* (d = .40, *p* < .001) and *safer sex norms* (d = .15, p = .022) and *safe sex attitudes* (d = .12, p = .016). No impact on safer sex intentions or perceived self-efficacy to engage in safer sexual behavior
Teadt et al. (2020) [23]	**Aim:** Scoping review for the use of New media platform for sexual health **Methods** • 16 studies were included (5 systematic review articles for social media, internet, web/based, social media TXs that included > 130 articles with African American youth)	• P: African American AYAs	• **Types of SRH TXs**: utilizing new media for improving contraception or condom use, communicating credible information regarding HIV and STIs, reducing the transmission of HIV and STIs, improving attitudes around sexual health, and promoting STI testing–related behaviors • **New media platforms** are defined as social networking sites, collaborative websites, blogs (for opinion sharing/discussion), content communities (for entertainment, info sharing), virtual reality/online gaming, communication/messengers. The most common forms utilized within the included studies were social media (e.g., Facebook, Twitter) (k = 10), internet-based interventions (e.g., *It’s Your Game-*Tech, *Keep It Up!*) (k = 5), mobile applications (k = 4), and interactive video games (k = 2)		**Feasibility of using new media for Reaching Youth & at-risk youth**: use media (Facebook) to reach a larger number of youth for sharing STI/HIV related info was highly feasible (85% viewing Facebook after invite; youth are highly motivated to access SRH info through new media given accessibility; marginalized groups were easy to engage in HIV care given user anonymity and less concern stigma/discrimination). ~ half of the studies (7/16, 44%) reported utilizing new media as an effective SRH promotion tool due to ease of use and wide accessibility in AYAs **Change in sexual health-related attitudes and behaviors**: several studies found the effects on sexual health knowledge, attitude/belief in delaying sex, self-efficacy in condom use, and having protective sex (e.g., one scoping review found an increase in the utilization of services/ # referrals/ testing; other study found 54% participants view STI info on Facebook as the most critical factor in their decision to change high-risk sexual behaviors)

*Note*. *AYA* Adolescents and young adults, *TX* Treatment/intervention, *STIs* Sexually transmitted infections, *AA* African American. *Eight best practices for app-based mHealth interventions for teen pregnancy prevention* suggested by Chen, et al. (2016) include [54]: (i) deliver and consistently reinforce persuasive communication about abstaining for sexual activity, (ii) about using condoms or other forms of contraception, (iii) based on theoretical approaches that have been demonstrated to influence other health-related behavior, (iv) provide clear, accurate information about the risk of pregnancy, (v) provide accurate information and skill-building exercises on how to use contraceptives to prevent unwanted pregnancy, (vi) provide skills-building exercise or practice with sexual communication, negotiation, and refusal, (vii) provide activities designed to engage users, personalized or internalize info, and provide tailored feedback, (viii) target information for special subgroups of adolescents (e.g., ethnic minorities, adolescents from LMICs)

**Table 3:** eHealth for GBV (All Age Groups) [59–62]

Article	Aim & Methods	Population (P)	Intervention (I)	Comparison (C)	Outcome (O)
Huang, et al. (2016) [61]	**Aim**: Review studies on sex-related apps and dating apps **Methods** • Free apps in the Apple iTunes store and Android Google Play store with English language interface were reviewed	• P for Sex apps: user reach between 1000 and 5000 (based on download records). Android apps that contained sexual health info were downloaded less frequently than other sex apps or entertainment apps • P for Dating apps: 73% apps (k = 44) target heterosexual users, 15% target men who have sex with men (MSM), 5% target lesbian women, and 7% for group dating	• Of the 137 sex-related apps, 15 (11.0%) had sexual health content, and 15 (11.0%) contained messages about sexual assault or violence. 78% of the apps did not contain any sexual health content • Of the 60 dating apps: Only 9 apps contained sexual health content, of which 7 targeted MSM	• Of the 15 apps that contained sexual health content, 33% had both contraception and STI info, 27% contained contraception info, and 40% contained STI and preventing STI info. Most apps were from the sex education and information category (73%) • Of the 15 apps offering information about sexual assault, 33% had info regarding management after sexual assault, 33% had general info about sexual assault, 7% (1) focused on identifying sexual assault victims, and the other 27% had information about sexual assault prevention. None mentioned the potential adverse sexual health outcomes faced by sexual assault victims	• Not studied
Anderson et al. (2021) [59]	**Aim:** A systematic review of Web-Based and mHealth TX for IPV **Methods** • 31 studies (23 unique TXs) published between 1998–2019 (61% published in or after 2015) • 67% RCTs or RCT protocols	• P: Adults or adolescents IPV victims (most ~ age 30) • 84% of studies included females; 85% IPV victims, 9% both victims & perpetrators, 9% pregnant victims; all victim-oriented TXs targeted only women • Countries: US (k = 23) & Cambodia (k = 1) • Settings: outpatient medical (26%), psychological/ therapy (10%), academic research (52%), community organization (13%) settings	• *mHealth TXs*: 29% web-based (WB) educational, 29% prevention outcomes depended on use of a computer, 19% WB interactive/responsive to participants; 13% WB communication w/TX provider • *Length of TX:* ranged from < 1 h to 14 weeks/6 month	• 26% of studies were computer-based screening with or without integrated education; 23% include safety decision aids. Most were secondary (n = 18) and tertiary (n = 10), and less were primary prevention (n = 3). Some IPV TXs were included as the secondary focus in the sexual health/violence-focused TX (n = 6) • Among RCTs, *control arms* included waitlist control, usual care, paper-based screening, face-to-face screening or TX delivery, health information materials related or not related to IPV	• Feasibility/acceptability are high • Insufficient evidence to support mHealth screening would increase IPV disclosure and better SRH outcomes (inconsistently defined) • IPV prevention with access to telehealth services (iCBT, online support groups for victims, changing behavior expectation through education) showed more promising in reducing IPV risk • Attrition is lower when using a WB method of participant-provider communication than the WB method without communication
El Morr et al. (2020) [60]	**Aim:** A systematic review of IPV, domestic violence TX that used ICT **Methods** • 25 studies addressing were identified • 64% (16/25) RCTs, 4/25 pre-post design	• P: Most are women (pregnant, postpartum, with a history of IPV, high HIV risk), except 1 includes both genders • Countries: 92% in North America, 12% Canada, 1 in Australia, and 1 New Zealand • mHealth provided in a medical setting (56%), community (24%), social service (16%), and legal service settings	• *IPV ICT TXs*: for screening, prevention, mental health TX, and support/empowerment • *ICT strategies*: 68% solely desktop or laptop-based, 8% tablet-based, 4% use tablet and telephone, 4% study implemented a kiosk system, 12% were not reported and supposed any type of ICT	• 52% studies were IPV screening and disclosure, 20% IPV prevention, 16% mental health TXs (e.g., video conference for IPV trauma); 8% support/ empowerment TX (e.g., enhance decision making & self-efficacy) • *Control arms* included usual care, face-to-face paper-based screening, wait-list control, non-tailored version of ICT, health information, interventions not related to IPV	• IPV screening/disclosure: equally effective or more effective as using the usual paper/face-to-face methods • IPV prevention: reduction on physical IPV, IPV injury, severe sexual IPV, physical aggression • Women’s mental health (TX): TXs that addressed mental health (MH) & substance use showed improvement on MH, PTSD & user satisfaction • Support/empowerment TXs: increase women's rate of leaving abusive partners, safety behaviors, improve decision-making skills, lower decisional conflicts • Usability of ICT software: generally are high
Linde, et al., (2020) [62]	**Aim:** Systematic review & meta-analysis of IPV eHealth TXs **Methods** • 14 RCTs in comparison to standard care, articles publish up till April 2019 were included (8 published between 2002–2019; 7 RCTs included in the meta-analysis; follow up from 1.5–12 months)	• P**:** Women exposed to IPV, and age ranged from 27 to 40 years • Countries**:** 6 US, 1 Australia, and 1 New Zealand	• *eHealth IPV TX*: IPV education, prevention, safety, support, skill-building • *mHealth strategies*: use of information and communication technologies for health, including mHealth, online WB platform, and telehealth	3 trials focused on Online safety decision aid (vs. control website or standard safety planning), 1 trial focused on *online IPV education* (vs online popular TV shows), 2 trials assessed *telephone support* (vs standard care), 1 trial focused on *email modules with relationship communication skills & problem-solving training* (vs. placebo email module), 1 3-armed trial focus *on email module with IPV support***(**vs. face-to-face modules with IPV support, vs Standard Care)	• Meta-analysis of RCTs found **no evidence** that eHealth TXs reduce IPV (overall IPV, physical violence, psychological violence, sexual violence, depression, and PTSD) • Subgroup analysis that compared low risk with high risk of bias trials showed similar results • Similarly, when data were stratified according to the type of scale or type of eHealth TX, subgroup analyses showed no effect of eHealth on the reduction of IPV

*Note*. *TX* Intervention, *WB* Web-based, *iCBT* internet-cognitive behavioral therapy, *ICT* Information and communication technologies, *MH* Mental health, *k* Number of study. *Info* Information

**Table 4:** eHealth consider adolescent developmental fit and needs [63–68]

**Article**	**Aim & Methods**	**Population (P)**	**Intervention (I)**	**Comparison (C)**	**Outcome (O)**
Lindsay et al. (2018) [64]	**Aim:** Systematic review of electronic mentoring TXs (for accessing social and peer support) **Methods** • 25 articles published between 1993–2018	• P: Children or youth aged 12–26 with disabilities • Countries: from 6 developed countries (14 US, 6 Canada, 2 Netherlands, 1 Australia, 1 Israel, 1 South Korea)	• **E-Mentoring delivery model**: The electronic mentoring interventions varied in delivery format and involved 1 or more of the following: interactive websites, virtual environment, email, mobile apps, Skype video calls, and phone calls. A total of 13 studies involved one-to-one mentoring, 6 had group-based mentoring, and 6 had a combination of both • **Types of mentors**: mentors who had a similar type of disability to the mentee (k = 12), mentors who were near-peers without disabilities (k = 2), and adult mentors without disabilities (k = 7) • **TX dosage**: The overall duration and dosage of the TXs reviewed ranged from 0.31 h per week to 2 h per week, occurring over a period of 4 to 24 weeks. Exposure ranged from 2 months to 4 years	• When compared with face-to-face mentoring, e-mentoring through interactive websites had similar outcomes for self-efficacy, quality of life, and self-management and for dealing with daily life • The benefits of e-mentoring programs for disability youth were similar to the youth without disabilities	• 11 studies testing significance, 9 (81%) reported a significant improvement in at least one of the following: career decision making, self-determination, self-advocacy, self-confidence, self-management, social skills, attitude toward disability, and coping with daily life • Positive effects of e-mentoring were reported for all types of mentors but, given the heterogeneity of outcomes, it was not possible to compare the effectiveness of types of mentors across studies
Low et al. (2019) [66]	**Aim:** Review Technology-based tools (web- or mobile-based TXs) for supporting AYAs health **Methods** • 29 studies (14 qualitative, 8 efficacy trials, 5 mixed methods) published between 2006–2009	• P: 7–28 years old (5 studies exclusive only adolescents). AYAs with chronic disease (e.g., diabetes, rheumatic disease, asthma, cystic fibrosis, hemophilia, et al.) • Countries: only developed countries (US, Canada, Netherlands, UK, Ireland, Australia, Germany)	• **eHealth TXs**: 13 via a website, 4 via mobile app • **AYAs preferred TX design**: using preexisting technology (e.g., mobile app) • **Preferred delivery method:** with visual appealing feature (graphics, games, audiovisual); learning through interactive games or watching educational short videos at a kiosk; tracking function, affordable/accessible, provide support • **Preferred communication strategies**: using peers to comment on the topic (e.g., disease management tips, transition/experience), updated research or disease news, and practical info (e.g., the difference between child and adult care, staff members). Mental health support was found to be an appreciated feature (e.g., manage anxiety, stress, IPV, alcohol use) (k = 3) • **Preferred Support from Peers or Health Care Professionals**: AYAs preferred an online support group (k = 3), network opportunity with peers (k = 7), online discussion forums (k = 4), using existing social media & gaining connection w health provider (k = 4)	• **eHealth design based on theory**: 8 TXs were based on theories such as Social Cognitive, Self-Efficacy, Self-Determination, and Social Learning Theory	• **Perceive usefulness or acceptability** (k = 16): Most TXs (k = 11) receive a positive reaction. AYAs were receptive to receiving medical information electronically • **Efficacy for web-based TXs** (k = 8): Meta-analyses showed no significant group differences across time on quality of life, self-efficacy, and self-management
Jeminiwa et al. (2019) [63]	**Aim:** Review of Theoretical Frameworks for evaluating the Quality of mHealth App for adolescent use **Methods** • 13 articles published between 2007–2017 • All studies included qualitative data (6 mixed methods, 6 qualitative)	• P: Target adolescent ages of 12–18 years and Normally developing • Countries: All developed countries (US, Canada, Germany, Ireland, UK)	• **Purpose of Apps**: Apps were designed for self-managing health (including asthma, cancer, type 1 diabetes, sickle cell disease, STIs, lupus, or mental health) • **App features preferred by adolescents:** Most commonly preferred features include the *ability to track results* or self-management progress, *connect to social media, offer peer support through social media***,** and *gain points or prizes* through app gamification	• Adolescents and adults differ in design preference • Adolescents *prefer networking with their peers*, or people of a similar age group, whereas adults prefer people with similar conditions, and trusted family and friends • For *provider support*, adolescents were primarily interested in providers’ ability to view health data for monitoring purposes and the ability to ask questions and receive feedback from providers	• **App Quality Rating Criteria:** Common rating criteria include the degree of app customizability, ease of use, visual appeal, interactivity (with peers, clinicians, or social media), and self-management capability • **The theoretical framework for Evaluating Quality of mHealth App: 5** dimensions emerged: 1) *Technical Quality (*including 8 constructs, such as app ease of use); 2) *Engagement* (including 6 constructs, such as app interactivity); 3) *Support System* (including 6 constructs, such as decision support, behavior change or learning support features); 4) *Autonomy* (including 3 constructs, such as app accessibility in terms of control, cost); and 5) *Safety, Privacy, and Trust* (including 3 constructs, such as app credibility, safety)
McCashin et al. (2019) [67]	**Aim:** Synthesize the literature regarding the experience of young people who have used technology-assisted Cognitive Behavioral Therapy (iCBT) **Methods** • 14 qualitative studies published between 2013–2018	• P: School-aged young people (> age 6 and < 18) • Countries: 6 New Zealand, 2 US, 2 UK, 1 Ireland, 1 Sweden, 1 South Africa, 1 Spain	• CBT is used for behavioral/emotional intervention. It considers the complex relationship between one’s thoughts, feelings, and behaviors (TFBs) in managing behavioral health. CBT provides a structured intervention that necessitates the use of metacognition (thinking about one’s thinking) to recognize problematic patterns adversely impacting the individual. [69] • For CBT to be effective with young people, it needs to be appropriately tailored to their developmental stage • **Technology-assisted formats of CBT (iCBT**) include internet-CBT, computerized-CBT/cCBT, CBT apps, CBT games, tele-CBT, and virtual reality CBT **iCBT included in this review** were TXs for mood & anxiety (k = 10), trauma or self-harm (k = 2), and physical difficulties (k = 2)	Comparing tech-assisted CBT with face-to-face CBT	**Tech-supported CBT is feasible for young people:** experience in 5 themes emerged (1) *helpfulness* (helpful, positive experience) (2) *therapeutic process* (CBT model can be understood, iCBT can guide cognitive-behavioral change and skills development) (3) *transferability of iCBT for young people in daily life* (activities can be applied to young people’s everyday life setting, and can be used by parents and children together) (4) *gameplay experience* (young people can be related to characters in the iCBT, and has good playability) (5) *limitations/negative experience* (insufficient to address self-harm thought/behaviors; broad/ generalized content may not be helpful; over-reliance on reading and writing skills)
Reen, et al. (2019) [68]	**Aim:** Review of health information website design for adolescents and synthesize the usability **Methods** • 25 studies published in English and between 2000 and 2019 that assess the usability of health info. websites on any health topic using survey or qualitative evaluation methods were included	• P: Age 11–25 years (mean age was 15.2 years). The majority were non-clinical population (79%) and some with disability (12%). Participants • Settings: recruited from medical/ clinical, school, juvenile justice, youth center, and community settings	• **Health Information Website**: designed to give general information for a combination of health topics (e.g., mental health, diet/weight management, drugs and alcohol, contraception, and sleep patterns) **Adolescent preferences of websites design–** • ***Appearance*** (visual appearance, organization) (k = 6): website with a simple layout and with color • ***Navigation*** (k = 10): easily navigate, simple log-in • ***Delivery of content* (**video, vignettes, animation) (k = 14): prefer short videos, images, audio clips, positive stories/ testimonials from other adolescents, and animations; content delivered to be easy to comprehend and through their peers • ***Message source*** (k = 3): prefer website with a clear logo and website name that is suitable to the target audience, and gender-balanced • ***Interaction & Participation*** (k = 18): prefer using games and quizzes, the ability to control/set goals or personalize the website, interacting with health professionals (for Q&A) or peers, social networking capability, and with incentive point-system	• **Comparing adolescents' & adults' preferences:** Adolescents’ prefer eHealth features are different from adults. The design needs to consider the context of adolescent social processes, low tolerance of delayed gratification, and attraction to novelty and fit with a neurodevelopmental model of adolescence	**Usability was assessed in multiple domains (in the reviewed studies):** appearance, navigation burden, content delivery methods, message source, interactive/participation function (see left column for design feature preferred)
Liverpool et al. (2020) [65]	**Aim:** Review models of delivery, facilitators, and barriers in engaging children and young people (CYP) in digital behavioral health TXs **Methods** • 83 articles (represent 71 mental health TXs)	• P: 2–24 years old CYP (46% with affect disorder,) • Countries: 37% study (k = 31) from US and Canada, 28% (k = 21) from Australia and New Zealand, 25% (k = 21) from Europe, 8% (k = 7) from Asian and 1 from Brazil	• **Model of delivery used in CYP digital behavioral health intervention:** 6 modes of delivery were identified: (**1) website** TXs (n = 43; 50% RCT) **(2) games and computer-assisted TXs** (n = 23; 50% RCT) **(3) Apps**: web or mobile (n = 10; 40% RCT), (**4) robots and digital devices** (n = 3; 33% RCT), (**5) virtual reality** (n = 3), and (**6) mobile text messaging** (n = 1, RCT) • **Communication and support eHealth strategies:** Website (email, text, social network, discussion forums, web-based message boards) • **Skill develop eHealth Strategies:** game or computer-assisted, Apps, virtual reality, text message • **Psychoeducation eHealth strategies:** games or computer-assisted (photos, stories, animations, quizzes, multimedia audio and videos) • **Information Dissemination eHealth strategies**: all 6 models of delivery can be applied	**Theory applied in Behavioral Health TXs design**: Cognitive-behavioral, cognitive skills training, social skills training/ social support, applied behavior analysis TXs **Retain rates by eHealth Delivery model**: the retention rates varied. The average retention rate for games and computer-assisted intervention studies was higher (86.95%), followed by websites interventions (78.87%), and apps (78.45%)	**Barriers and Facilitators to engaging CYP in digital behavioral health:** • ***Person-specific features preferred:*** feeling a sense of person connectedness (connection to others with similar experience), sense of trust (trusted brand names, from a credible source, anonymity), motivating usage (elicit curiosity and perceive needs, sense of helpfulness influence usage) • ***Intervention-specific features preferred*:** CYPs suggested features such as videos, having less text, ability to personalize or create a profile, ability to connect with others or receive text message reminders, providing a reward, self-paced, simple/easy understand/straightforward, age-appropriate, user-friendly, fit lifestyle as encouraging their use of the intervention

*Note*. *K* Number of studies

### Findings around digital health for adolescent SRH promotion

Table 2 summarizes SRH digital health intervention studies for adolescent and young adults’ (AYAs’) SRH. Across 7 review studies included in our review, 154 articles and 22 apps were included in the review articles. *Overall****,**** we found that most digital SRH interventions focused on both adolescents and young adults (not adolescent-specific), and use diverse digital strategies (text message, social media, interactive strategies) for SRH screening, knowledge promotion, and behavioral intervention. Although several high-quality apps are available, the evidence for promoting SRH is mixed. More theory-guided digital interventions (cognitive, behavior, or ecological framework) and systematically testing digital strategies are needed to improve the impacts.* Here we summarize the key findings.

#### Targeted populations

The populations included in this area of research were all targeted on adolescents and young adults (see Table 2 for age ranges defined in the reviews), and included quite diverse populations (e.g., African American, Latino, LMIC populations, both gender), with one review study focused on adolescents exclusively from eight LMICs [56]. Although diverse populations are considered, most studies did not examine digital health use and impact on SRH separately for younger and older AYAs.

#### Topics of intervention

United Nations Family Planning Association (UNFPA) suggests covering *9 topic areas in SRH* services (described above) [2]. From our review, we found the digital approach of SRH interventions in AYAs were mainly focused on contraception, pregnancy prevention, abstinence promotion [23, 54, 56–58], sexual health promotion (on topics related to STIs, HIV/AIDS, safe sex, risky sex behaviors) [23, 53, 55–58], substance use [53], family planning counseling, abortion care [56], and GBV [56]. The existing AYA e-SRH interventions cover 5 of the 9 SRH areas suggested by the UNFPA, but predominately focus on pregnancy prevention, uptake of contraception, STIs and HIV/AIDS prevention, and treatment referral and follow-up. Only a small aspect of GBV/IPV was included as part of SRH services.

#### Digital health strategies & functions included

Three themes emerged. For the primary prevention focused *SRH interventions*, greater emphasis is on using technology to promote knowledge, healthy SRH behaviors (e.g. behaviors for preventing pregnancy), and STI screening and follow-up (through public health campaigns or individual outreach). Some interventions also provide counseling or referral support to link users to essential SRH services when needed (e.g., medically assisted abortion, family planning, STI, HIV care).

There is a wide range of *digital health strategies* applied in SRH promotion and knowledge dissemination, but the use of technology strategies seems to shift overtime. For example, *mobile phone and text messaging* were the most frequent options for the study conducted before 2015 [53, 56, 57]. Recent studies have integrated more *social media-based strategies* (e.g., internet-based social media Facebook, WhatsApp, Twitter, Snapchat, Instagram, blogs, virtual reality, online gaming) [23, 55], and promoted *more interactive technology* (e.g., accepting input from the users, interactive video games) *or technology with tailored functions.* Some studies suggested that these new social media strategies can be effective and efficacious in rapidly promoting attitude and norm change around SRH (e.g., promoting safe sex norm, attitude, and condom use attitude) [58]. More research is needed to better understand whether different technology strategies are associated with different levels of engagement and harm/safety issues, which impact AYAs’ SRH outcomes [56].

There are several *high-quality mobile apps* (*my choice by PPT*, *Safe Sex Tips*, *Get S.M.A.R.T*) developed for adolescent pregnancy prevention. These apps were based on recommended best practice guidelines for pregnancy prevention (see Notes under Table 2 for the best practice guideline) and credible/reputable public health information sources (e.g., US Center for Disease Control/CDC) [54]. The availability of these apps is encouraging, but more evidence for underline mechanisms is needed to understand how apps designed based on best practice guidelines impact short and long-term SRH outcomes [54].

#### Feasibility, efficacy, effectiveness evidence and gaps

*The impact evidence* on technology-based SRH promotion is encouraging but mixed. Some general patterns can be drawn from the literature. In *sexual health promotion*, *online social media* (Facebook) and *other technology-based strategies* (computer, texting, web-based, internet-based) have been reported with small but significant positive impacts (*d* = 0.21-0.26), especially in changing knowledge, negative attitudes, and promoting healthy sexual behaviors (condom use, abstinence, STI screening/follow-up) [23, 55, 58, 70]. These findings tend to be similar by age, gender, country, and intervention dose, and tend to be short-term (not maintained over 6 months) [55, 58]. In *SRH promotion*, studies using *texting and mobile phone strategies* [53, 54, 57] have found that texting alone tends not to be sufficient to improve knowledge and practice outcomes of SRH. The combination of text messaging with other strategies (e.g., adding behavioral change strategies/curriculum, financial incentives, educational story-telling, video messaging, psychosocial support/counseling, Q&A bidirectional texting, including use of screening) are more likely to yield higher rates of screening, follow-up, and positive SRH outcomes (knowledge, awareness, unprotected sex, condom use, STI testing) [56, 57]. Future research is needed to test cognitive (knowledge/attitude/belief) and behavioral change theories to study intervention effectiveness mechanisms on sexual health and reproductive health knowledge and practice uptake and maintenance for adolescents and young adults separately given different developmental ability and characteristics [56].

### Findings around digital health for GBV prevention

Table 3 summarizes digital health for IPV and GBV for all age groups. Across 4 review studies that we reviewed, 70 digital health articles and 197 applications (apps) in the Apple iTunes and Android Google Play stores were reviewed. *Overall, we found that most digital GBV including IPV interventions have focused on female and high-income country populations. Most digital GBV/IPV interventions were focused on secondary or tertiary interventions (not primary prevention) and included diverse digital health strategies (apps-, web-, and/or mobile-based communication strategies). Although feasibility and acceptability of using digital health for GBV/IPV screening and interventions are generally high, there is insufficient effectiveness evidence to support that use of digital health (targeting GBV/IPV risks) would lead to better SRH outcomes. More integrated intervention research (target on multiple risks) and comparative effectiveness research are needed to document how the combination of intervention strategies improves GBV/IPV and SRH outcomes.* Below we summarize results in 6 areas.

#### Targeted populations

The population that most IPV and GBV digital health interventions targeted were women and IPV survivors from high-income countries. Very few studies targeted men or perpetrators [59, 60, 62] or populations in LMICs. No digital health study targeted adolescents specifically or addressed GBV issues. This explains why, adolescent-specific digital health guidelines and strategies to prevent and management of IPV and GBV are not in place.

#### Topics of intervention

Specific to *IPV the digital health interventions* that have been developed are for IPV screening and disclosure, prevention and education, mental health interventions, empowerment, and social support purposes. However, most technology-based IPV interventions were secondary or tertiary interventions [59, 60], and only a few were focused on primary prevention. Lack of IPV digital health research in adolescents may explain why IPV and GBV services have not been included [62].

#### Digital health strategies/functions included

Digital health strategies that have been applied in IPV interventions include: using apps and web-platform for education, using a web or mobile-based communication (including video conferment/telehealth, email), and interaction functions to engage and address participants’ needs. Most IPV interventions apply multiple digital strategies [59, 60, 62]. Open-source apps tend to be designed for sex aid/sexual exploration and entertainment purposes, and are less likely to be used for IPV education [61].

#### Feasibility

Across studies, *feasibility, and acceptability* of using digital health for IPV screening and interventions are generally high, but questions addressing safety, equity, and the unintended consequences of the use of digital health in IPV programming are virtually non-existent [60].

#### Efficacy and effectiveness evidence and gaps

Although some impact evidence suggests that e-IPV interventions could improve understanding of how and why violence occurs, provide knowledge on disclosure, safety, and management skills [60], there is insufficient efficacy or effectiveness evidence to support that IPV digital health would lead to better SRH outcomes [62, 71]. There is also a lack of comparative effectiveness research between digital health and the more conventional IPV and GBV intervention approaches. Outcome comparisons across studies are difficult given standardized measures of SRH, IPV, and GBV outcomes were not used across studies and quite different IPV and GBV intervention contents were included. More research using well-defined and standardized measures is needed.

IPV prevention (especially commonly used educational programs) and screening alone are not sufficient to prevent or intervene for IPV. Inclusion of additional intervention strategies such as improved access to digital health support services (e.g., telehealth, patient-provider e-communication, online support groups, technology-based cognitive-behavioral-therapy/CBT) are more likely to show promising outcomes in reducing IPV risks while promoting SRH. [59]. This suggests that most current SRH programs that include educational material only for the positive and respectful approach to sexuality and sexual relationship might not be sufficient to meaningfully address adolescents’ IPV and GBV needs.

### Findings around digital health for adolescent development promotion

Table 4 summarizes digital health applications in promoting adolescent and young people's health and development. Six review studies were included for this review (representing 189 digital health articles). In this category of literature, two review papers focused exclusively on children and adolescents below age 18 (6–18 years old) and considered their developmental characteristics in digital health design and feedback query processes [63, 67]. Remaining four review articles included both adolescents and young adults. *Overall, we found adolescents differed from adults in digital health design preference (interactive/ participatory function). Digital health interventions (web-based and app-based interventions) have been applied for psychoeducation and skill development purposes. User-centered design studies have informed the development of several digital-health design guidelines for teens and adolescents. Although evidence has shown feasible of digital health for adolescent health promotion, effectiveness evidence for digital health remains to be limited.* Below we summarize results in 6 areas from this area of literature.

#### Characteristics of the Targeted populations

Adolescent users have unique features and preferences in digital health use. Designing digital health solutions requires careful consideration of their life context, lifestyle, their social processes, low tolerance of delayed gratification, and attraction to novelty [63, 65–68]. Adolescents also prefer networking opportunities and gaining connections with health providers [66].

#### Topics of intervention and digital health strategies

mhealth apps and web-based *interventions* are commonly applied to promote adolescent health literacy and health behaviors. A body of user-testing/feedback studies with adolescents has informed the development of several useful frameworks/guidelines for guiding the design of the mHealth apps for adolescent use [63, 68]. For example, Jeminiwa and colleagues proposed a five-dimensional *framework for guiding the design and evaluating the quality of mHealth apps for teens and adolescents*. The 5 dimensions include considering the technical quality (such as app ease of use), engagement (such as interactivity), support system (such as decision support, behavioral or learning support feature), autonomy (such as app accessibility in terms of control and cost), and safety, privacy, and trust (such as app credibility, safety) [63]. Reen and colleagues [68] proposed another five-dimensional *framework to guide health information website design for adolescents*. Reen et al. suggested that a considered website design in the following 5 areas, as well as user suggested strategies in each area, is important (listed below): *i) appearance* (website with a simple layout and with color), *ii) negative burden* (easy navigation, simple log-in), *iii) content delivery methods* (adolescents prefer short videos, images, audio clips, positive stories/testimonial from other peers, animation; content delivered to be easy to comprehend and through peers), *iv) message source* (website with clear logo and appropriate website name, gender balance), *v) interactive/participation functions* (prefer using games/quizzes, ability to control/personalize, to interact with professionals or peers, and incentive point-system, social network capability) [68].

Depending on the goal of digital health intervention (for skill development or knowledge promotion), different *digital health design and implementation strategies* may be applied. For *skill development*, digital health strategies such as using game/computer-assisted, apps, virtual reality, text messages can be very relevant. For *psychoeducation purposes*, games or computer-assisted solutions (using photos, stories, quizzes, multimedia audio/video) can be optimal options [65].

#### Feasibility

It is feasible to apply digital health for adolescent health promotion. Specifically, cognitive-behavior therapy (CBT) is an intervention approach that helps people cope with stress or emotion-behavior challenges. Technology-assisted formats of CBT (iCBT) have been adapted for children and adolescents and have shown to be a feasible approach to help young people manage their emotional stress [67].

#### Efficacy, effectiveness evidence and gaps

E-mentoring has been recommended as an effective way to support adolescents who face multiple challenges. E-mentoring can be flexible and use multiple existing technologies to support (video call, email), and can be equally effective as face-to-face mentoring. E-mentoring can promote self-confidence, skill development, and coping with daily life [64]. Evidence from e-mentoring can be relevant to SRH promotion.

Most digital health review studies included in this developmental category have been focused on *identifying effective, safe, trusted, engaged, and developmentally appropriate digital health models of delivery and strategies* that fit different developmental stages preferring more qualitative and mixed-methods types of study designs. They have not focused on effectiveness evaluation (given multiple health topics were considered).

## Discussion

Our umbrella review of three inter-related areas of research has provided deeper insights to understand current challenges in advancing short and long-term SRH and socioemotional development in young people living in LMICs. In each area of review, we focused on synthesizing findings for 6 areas of questions: (i) which populations and users have been targeted in the digital intervention research; (ii) what areas of SRH topics have been covered in the digital health interventions; (iii) what technology platforms/application and digital implementation strategies have been used in these studies; (iv) whether/what digital health strategies are acceptable and feasible; (v) what impact evidence and related recommendations have been documented from the digital intervention studies; and (vi) which under-studieddigital intervention research strategies need to be prioritized in future research. Our review contributes to new knowledge in applying technology and ICT strategies for SRH and GBV/IPV intervention and implementation strategy.

*In the digital SRH research*, we found most interventions have focused on applying intervention delivery models and strategies that have been tested in adults and applied similar intervention models without differentiation to adolescents. Adolescent developmental characteristics and needs (e.g., emotion, cognitive comprehension, material, identity, both peer and family support needs) have rarely been discussed in digital SRH design or for example in reproductive care for pregnant adolescents. Similarly, *for the digital GBV and IPV research*, we found most solutions have focused on secondary and tertiary interventions in women, victims, and adults. The technology approach of GBV interventions have not been offered from preventive and skill-building perspectives or considered targeted adolescents such as younger-aged boys and girls, and altogether these have not focused on beliefs/attitudes and behavioral change as outcomes of interest. *In the field of adolescent development research*, we found great progress has been made in identifying developmentally appropriate and engaged digital health strategies and platforms for different health promotion goals (e.g., education/knowledge learning, communication, mentoring, support, information dissemination/sharing, health behavioral change). These user-centered design thinking and guidelines are useful for future digital SRH, and GBV/IPV intervention design and testing. Overall, across our highlighted areas of literature, consistent findings on high feasibility and acceptability using digital health strategies for adolescent SRH, IPV, GBV, and development promotion have been documented. However, effectiveness research evidence for digital health intervention is insufficient and mixed, especially for tracking broad health and long-term SRH outcomes. More adolescent population-specific e-intervention evidence is urgently needed.

### Recommendation for integrating GBV-SRH-developmental service for adolescents in LMICs

To identify strategies and draw recommendations for integrated GBV/IPV prevention within SRH services that fit the development of adolescents, we synthesized our review observations and our group’s adolescent health research experience to further identify opportunities and strategies which hold promise. As authors reviewed the findings and have generated key recommendations. Below, we provide five sets of recommendations that hold promise to developing integrated services.

#### Starting from youth service provider education and community awareness campaigns

The current GBV/IPV, and SRH service integration gaps can be attributed to a lack of knowledge and inadequate skills in addressing inter-related adolescent health issues in service providers, school staff, and parents/caregivers of adolescents. Several digital health strategies identified from this review are promising and can be applied for *education purposes* (such as web-based training modules, virtual video, simulated videos), *decision-support purposes* (e.g., tablet-based screening, decision support for interventions), and *information dissemination/awareness campaign purposes* (e.g. social media). Some IPV studies have focused on training providers (e.g., midwives) to provide tablet-based IPV screening and detection, and early intervention to promote safe behaviors in peripartum women and adolescents [72]; or to use simulated nursing video consultations to train nurses in GBV clinical management or referrals (which also improved nurses’ GBV awareness and victim management knowledge and skills) [73]. Applying similar digital health strategies to improve adult caregivers, primary care staff, school staff, and community youth service providers’ knowledge and competency in supporting adolescents’ GBV, IPV, SRH, and development can be prioritized.

#### Providing integrated SRH service by building on the existing recommended high-quality e-SRH tools and strengthening the existing e-SRH service platforms

Multiple service settings (i.e., medical/clinical, community service, social service, juvenile justice, youth center, schools, legal service, homes), and existing e-SRH platforms (web-based, internet-based, mobile-based, new media) have been set up for providing SRH, GBV, and developmental preventive services. There are also high-quality SRH apps/tools that are based on the best practice guidelines (e.g., *my choice by PPT*, *Safe Sex Tips*, *Get S.M.A.R.T*) developed. Therefore, strengthening the capacity of the existing e-SRH delivery system and platform to further provide age-appropriate and evidence-informed GBV (including IPV) services can be a potentially cost-effective strategy to offer integrated care. As discussed above, GBV are complex social problems that can be attributed to multi-level factors. When strengthening the existing e-SRH system, additional digital strategies for social norm change (e.g., social media), e-psychosocial support and professional support strategies (e.g., Q&A support strategies) and service linkages should be integrated as well in order to set up a pathway of care from preventive to referral and treatment services.

#### Applying multiple technology strategies based on the feasibility and user-feedback findings

Across the reviewed literature, there is a consistent finding reporting high feasibility and acceptability towards integrating digital health strategies for adolescent SRH, GBV/IPV and development promotion. *In AYAs’ sexual health promotion*, it has been found that online social media (Facebook) and other technology-based strategies (computer, texting, web-based, internet-based) resulted in meaningful short-term impacts in changing knowledge, negative attitudes, and by promoting healthy sexual behaviors. In the *field of SRH promotion*, it has been found that a combination of *using texts and mobile phone strategies* with other digital health strategies (e.g., adding behavioral change strategies/curriculum, financial incentives, Q&A bidirectional texting) has the potential to yield higher rates of screening, follow-up, and positive SRH outcomes. *In the adolescent development field*, several digital health strategies have been identified to be useful for *skill and healthy behavior development* (e.g., using a game, apps, virtual reality, text messages, technology-assisted formats of iCBT), for *psychoeducational purpose*s(e.g., games or computer assist using photos, stories, quizzes, multimedia audio/video, E-mentoring via video call, email using peer-to-peer or adult-to-adolescent), and promoting adolescent self-confidence, skill development, and coping with daily life stressors. These need to be further optimized. Building on this evidence and further studying feasible strategies with a diverse group of adolescents in LMICs using standardized outcome measures will be an important next step to generate effective digital health evidence for adolescent health and development.

#### Engaging adolescents from diverse backgrounds for testing integrated e-SRH service design and dissemination will lead to greater insights

Learning from findings around adolescent development, we found adolescents are different from adults with regards to their digital health design preferences. They prefer certain digital health delivery methods, communication strategies, engagement approaches, and ways for gaining support from peers and health care professionals. Additionally, we also identified that digital health designs need to fit with the lifestyle of adolescents in order to have greater traction and uptake. Therefore, integrating e-SRH, and GBV services need to consider the needs and lifestyle of vulnerable sub-population, including pregnant and non-pregnant adolescents, boys and girls, and school and out-of-school adolescents. Engaging adolescents from these diverse groups in design, dissemination, and service provision processes can lead to enhanced user-centered, integrated e-SRH care that can speak to the diversity of stakeholders that need to be considered for the advancement of the field [70, 74].

#### Providing the integrated e-SRH preventive service in diverse primary care and community settings using multiple digital health strategies to reach non-clinical populations

SRH and GBV are considered as common health and developmental health topics that need to be addressed through universalizing preventive care. However, the existing GBV interventions tend to focus on IPV, victims and women/girls, but do not target men/boys or lower-risk adolescents who need empowerment and skills in time. To have greater population reach in SRH promotion and GBV prevention, schools and community organizations can become ideal settings to provide integrated preventive e-GBV- SRH solutions and skills, especially for adolescents. Future research can engage adolescents and service providers from these settings to further test appropriate digital health strategies. This may include using mobile- or internet-delivered self-guided digital health models or cognitive behavior therapy (iCBT) for GBV that incorporated emotion-regulation/social emotion skill development and conflict-resolution techniques in combination with other e-support strategies [75].

In summary**, o**ur study is the first to integrate multiple areas of digital health intervention literature. Our paper is an attempt to identify strategies to improve quality and age-appropriate SRH services for adolescents in LMICs. Although this work has contributed to new knowledge, one key limitation should be kept in mind. Specifically, we did not conduct an extensive systematic review. Therefore, some digital health studies for adolescents may be missing (e.g., digital health intervention research for adolescents).

This review was not without its limitations. The use of the one databased (PubMed) might not eliminate databased bias issue. Our choice of search terms (i.e., eHealth and mHealth) might exclude some digital health studies. Finally, we have used an umbrella review strategy, while that adds strength to our overall findings primary studies have not been looked at for rapid evidence synthesis. The review was carried out in 2021 and the findings of this work need to keep in mind that the field has moved since then. However our broad strategies and recommendations we think still apply. Our search terms may not have captured all aspects of SRH especially the reproductive health and rights in particular. Given that the focus was on youth we think we may have still captured broad domains.

## Conclusion

We reviewed digital health intervention literature in SRH & GBV specifically focusing on IPV, and adolescent development promotion and prevention fields to identify models of delivery, digital health intervention designs, and promising digital health strategies. Our umbrella review underscores that applying digital health strategies (from traditional text messaging to more recent diverse media approaches) for adolescent health and development promotion is a highly recommended, feasible and acceptable solution. Our paper has identified many useful digital intervention and technology strategies to improve quality and age appropriate SRH services for use with adolescents in LMICs. Although effectiveness research is insufficient to make strong practice recommendations, user-center design guidelines are well-developed for web- and app-based digital health design for use by adolescents. We were also able to identify strategies, digital health tools, and digital health platforms that can be leveraged to further develop an integrated GBV-SRH-development service. We conclude by providing an Adolescent-Centered Integrated SRH Intervention conceptual framework and offering five sets of key recommendations for guiding future integrated SRH, GBV/IPV prevention and developmentally appropriate services in LMICs. Continued evidence generation with a focus on multiple digital health strategies along with standardized outcome measurement in adolescents and sub-adolescent populations are highly recommended. Alongside we urge use of longitudinal experimental designs that will be crucial in the generation of evidence-based interventions and practice guidelines for emerging needs of adolescents in resource constraint regions.

## Data Availability

Not applicable. This paper reviewed existing peer-reviewed articles. All papers reviewed in the study can be accessed through health science journal publication sources.

## Abbreviations

LMICs: Adolescents in low-and-middle-income countries
SRH: Sexual and reproductive health
GBV: Gender-based violence
IPV: Intimate partner violence
FGMC: Female genital mutilation/cutting
ICT: Information and communication technology
SSA: Sub-Saharan Africa
PICOS: Population targeted, Intervention characteristics, Comparison, Outcome, and Study type
CBT: Cognitive behavior therapy
